# The Development of a Novel mHealth Tool for Obstructive Sleep Apnea: Tracking Continuous Positive Airway Pressure Adherence as a Percentage of Time in Bed

**DOI:** 10.2196/39489

**Published:** 2022-12-05

**Authors:** Angela Fidler Pfammatter, Bonnie Olivia Hughes, Becky Tucker, Harry Whitmore, Bonnie Spring, Esra Tasali

**Affiliations:** 1 Department of Preventive Medicine Feinberg School of Medicine Northwestern University Evanston, IL United States; 2 Department of Medicine University of Chicago Chicago, IL United States

**Keywords:** obstructive sleep apnea, continuous positive airway pressure, CPAP adherence, weight loss, lifestyle

## Abstract

**Background:**

Continuous positive airway pressure (CPAP) is the mainstay obstructive sleep apnea (OSA) treatment; however, poor adherence to CPAP is common. Current guidelines specify 4 hours of CPAP use per night as a target to define adequate treatment adherence. However, effective OSA treatment requires CPAP use during the entire time spent in bed to optimally treat respiratory events and prevent adverse health effects associated with the time spent sleeping without wearing a CPAP device. Nightly sleep patterns vary considerably, making it necessary to measure CPAP adherence relative to the time spent in bed. Weight loss is an important goal for patients with OSA. Tools are required to address these clinical challenges in patients with OSA.

**Objective:**

This study aimed to develop a mobile health tool that combined weight loss features with novel CPAP adherence tracking (ie, percentage of CPAP wear time relative to objectively assessed time spent in bed) for patients with OSA.

**Methods:**

We used an iterative, user-centered process to design a new CPAP adherence tracking module that integrated with an existing weight loss app. A total of 37 patients with OSA aged 20 to 65 years were recruited. In phase 1, patients with OSA who were receiving CPAP treatment (n=7) tested the weight loss app to track nutrition, activity, and weight for 10 days. Participants completed a usability and acceptability survey. In phase 2, patients with OSA who were receiving CPAP treatment (n=21) completed a web-based survey about their interpretations and preferences for wireframes of the CPAP tracking module. In phase 3, patients with recently diagnosed OSA who were CPAP naive (n=9) were prescribed a CPAP device (ResMed AirSense10 AutoSet) and tested the integrated app for 3 to 4 weeks. Participants completed a usability survey and provided feedback.

**Results:**

During phase 1, participants found the app to be mostly easy to use, except for some difficulty searching for specific foods. All participants found the connected devices (Fitbit activity tracker and Fitbit Aria scale) easy to use and helpful. During phase 2, participants correctly interpreted CPAP adherence success, expressed as percentage of wear time relative to time spent in bed, and preferred seeing a clearly stated *percentage goal* (“Goal: 100%”). In phase 3, participants found the integrated app easy to use and requested push notification reminders to wear CPAP before bedtime and to sync Fitbit in the morning.

**Conclusions:**

We developed a mobile health tool that integrated a new CPAP adherence tracking module into an existing weight loss app. Novel features included addressing OSA-obesity comorbidity, CPAP adherence tracking via percentage of CPAP wear time relative to objectively assessed time spent in bed, and push notifications to foster adherence. Future research on the effectiveness of this tool in improving OSA treatment adherence is warranted.

## Introduction

Approximately one-third of the world’s population is considered overweight or obese [1]. Obesity is a major risk factor for obstructive sleep apnea (OSA), a sleep disorder characterized by recurrent complete or partial upper airway obstruction that results in reduced oxygen levels at night, sleep fragmentation, and poor sleep quality [2]. OSA is a global public health and economic burden, estimated to affect one billion people worldwide [3-5]. Untreated OSA is commonly associated with daytime sleepiness and neurocognitive impairment, which increase the risk of motor vehicle accidents [6]. Furthermore, there is strong evidence that beyond the effects of excess weight, OSA is associated with increased cardiometabolic risk and all-cause mortality [7-9]. Currently, continuous positive airway pressure (CPAP) applied at night is considered the treatment of choice for OSA, and there is no US Food and Drug Administration–approved drug treatment for OSA [10]. CPAP works by delivering continuous air pressure and preventing upper airway closure during sleep. It can be easily applied using a variety of masks worn on the face at night and is highly efficacious in treating OSA. However, poor adherence to CPAP therapy is a common problem [11-13]. CPAP adherence is defined as CPAP use for >4 hours per night; evidence from clinical research studies and real-world data suggests that adherence is variable among individuals, with a large proportion of patients being nonadherent to the treatment [13-16]. Several interventions such as educational materials, motivational interviewing, remote monitoring, and mobile health (mHealth) technologies have been used to promote adherence to CPAP therapy but have provided limited clinical translation to routine patient care [11,17-19]. For implementation in clinical practice, interventions aimed at fostering treatment adherence should be cost-effective and scalable to large and diverse patient populations. Therefore, novel approaches are urgently needed to promote adherence to CPAP therapy. In addition, weight loss is often recommended for patients with OSA, but it remains a major challenge in this patient population [20,21]. Thus, there is a critical need to develop new tools to address these important clinical barriers in OSA management.

Effective OSA treatment requires all-night CPAP use, that is, 100% of the time spent in bed, to optimally treat respiratory events, hypoxia, and sleep fragmentation and thus prevent adverse health effects associated with hours slept without wearing a CPAP device. Therefore, an accurate calculation of adherence to CPAP use requires a “denominator,” that is, hours spent in bed, which differs from adherence to, for example, medication use. However, current CPAP adherence tracking systems (eg, smartphone apps) simply capture the number of hours the CPAP device is used per night but do not account for hours spent in bed without using the CPAP device [22]. Moreover, in clinical practice, according to Medicare criteria, patients who wear their CPAP device for ≥4 hours per night for 70% of the nights are considered “adherent” to therapy. These policy recommendations also have implications on health equity, given the known racial or ethnic and socioeconomic differences in sleep patterns, particularly sleep duration [23]. The use of 4-hour CPAP wear as a cutoff point defining adequate treatment adherence is arbitrary and could be misleading because sleep patterns can vary considerably from night to night and among individuals, making it necessary to measure CPAP adherence relative to time spent in bed. For example, based on a 4-hour CPAP adherence threshold, a patient who uses a CPAP device for 5 hours but spends 8 hours in bed per night would be considered adherent. However, the patient’s true CPAP adherence should be only 63% as a percentage of time spent in bed. Hence, the patient’s treatment adherence should be considered suboptimal, and the patient should be advised clinically to increase their CPAP use. Implementing more correct CPAP adherence goals and guidelines requires quantification of CPAP wear time in proportion to the time spent in bed. In this regard, wearable mHealth devices offer a promising tool for at-home monitoring of sleep using accelerometry-based technology [24-26]. Combining such mHealth technology with CPAP use data provides a unique opportunity to revolutionize OSA treatment adherence guidelines and fulfill a critically unmet need for patients and health care providers.

Our goal was to develop a customized mHealth tool to support treatment adherence to both CPAP and weight loss recommendations in patients with OSA. We developed and tested a unique CPAP adherence tracking module that measured CPAP wear in proportion to the time spent in bed. We aimed to integrate this new CPAP adherence tracking module into our previously developed mHealth technology targeting lifestyle behaviors (nutrition and physical activity) to achieve weight loss in the population with OSA. This is a formative work that used an iterative, user-centered process to design a new CPAP adherence tracking module that integrated with an existing weight loss app; thus, the effectiveness of this tool in treatment adherence has not been tested or reported.

## Methods

### Overview

This study was conducted between January 2020 and March 2021. This study leveraged our existing technology platform for delivering smartphone apps to patients and web-based dashboards to interventionists [27]. The platform was built specifically to support behavioral interventions [27-29] and has the flexibility to display participant- and coach-facing features according to specifications such as the research study to which they belong and the behaviors targeted for change. Existing participant-facing smartphone apps include features for behavior change interventions to foster weight loss, healthier diet quality, physical activity, and smoking cessation.

Through an iterative user-centered design process, we developed and tested a new CPAP adherence tracking module that integrated our existing weight loss app with nutrition, activity, and weight tracking features. Our platform combined information from multiple devices, including Fitbit activity trackers and Fitbit Aria scale using the Fitbit application programming interface (API) and CPAP devices (ResMed AirSense 10 AutoSet) using the AirView API. In addition, we refined a companion interventionist web-based dashboard [28] to present interventionists with relevant information from the new CPAP adherence tracking module and the integrated diet, activity, and weight tracking features that they use to tailor coaching.

### Iterative User-Centered Design Process

**Figure 1:**
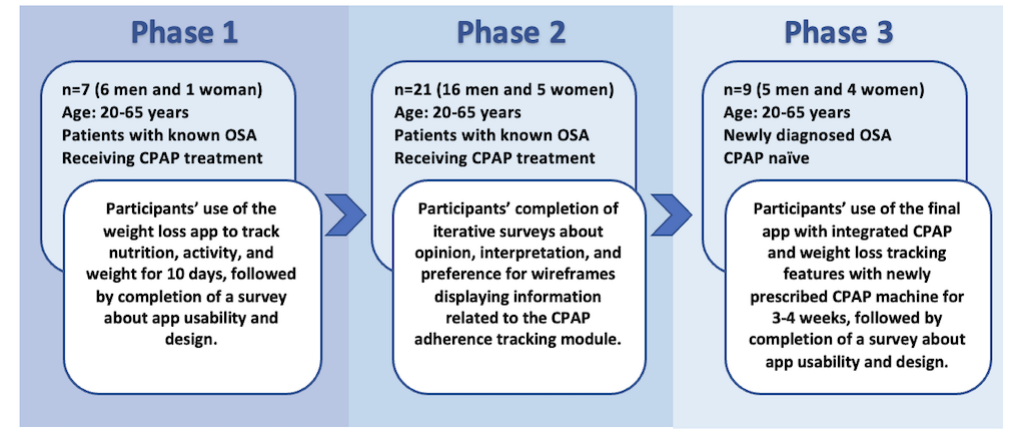
Study design and participant characteristics for each study phase. CPAP: continuous positive airway pressure; OSA: obstructive sleep apnea.

A 3-phase iterative user-centered process was implemented to develop a new CPAP adherence tracking module that integrated our existing weight loss app. Figure 1 provides details of the study design methods and participant characteristics for each study phase. Adult men and women were recruited according to inclusion criteria of age (aged 20-65 years) and an OSA diagnosis. There were no exclusion criteria based on BMI, race or ethnicity, or other demographic characteristics.

In phase 1 (mean age 45, SD 8 years), we aimed to collect feedback on the existing weight loss app from patients with known OSA who were receiving CPAP treatment. Patients who returned for a follow-up appointment at the University of Chicago Sleep Disorders Clinic were recruited if they had a prior diagnosis of OSA and a previously prescribed CPAP device. Potential participants were given a study flyer by their treating physician during clinic visits. Interested individuals discussed the study details with the study coordinator and were enrolled in the study after obtaining informed consent. The participants tested the existing weight loss app along with connected devices, including a wrist-worn Fitbit activity tracker (Fitbit Inspire HR) and a Fitbit weight scale (Fitbit Aria), to self-monitor and receive feedback on their dietary intake, physical activity, and weight for 10 days. At the end of the 10-day period, they completed a survey about the app design that included the System Usability Scale [30], a measure of usability on a 0- to 100-point scale where 65 is the threshold for a system to be considered usable. Participants were compensated US $100 for participation.

**Figure 2:**
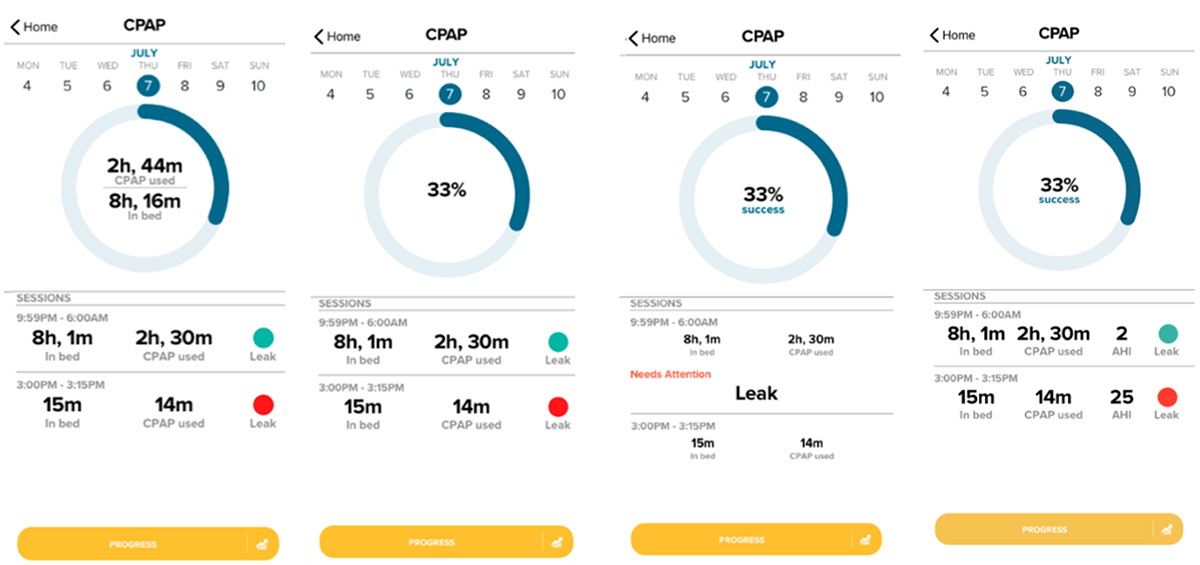
Example wireframes displaying continuous positive airway pressure tracking module shown to participants by web-based surveys.CPAP: continuous positive airway pressure; AHI: apnea-hypopnea index.

In phase 2 (mean age 47, SD 9 years), patients with known OSA who were receiving CPAP treatment were studied. Patients who returned for a follow-up appointment at the University of Chicago Sleep Disorders Clinic were recruited after obtaining informed consent if they had a prior diagnosis of OSA and a previously prescribed CPAP device. The participants completed a web-based survey to provide their preferences for graphical displays and interpretations of various wireframe images displaying information from the CPAP tracking module. Figure 2 illustrates examples of the wireframes shown to the participants. Using the various metrics of CPAP use that are available via the AirView API, the phase 2 of the study aimed to develop a customized CPAP adherence tracking module that displayed information deemed most relevant and helpful by patients receiving CPAP treatment. Participants were asked to describe their understanding of the information displayed on each screen (eg, percentage adherence—percentage CPAP wear time relative to objectively assessed time spent in bed; mask leak; and apnea-hypopnea index [AHI], that is, number of respiratory events per hour of sleep) and how they would react or change their CPAP use behavior after seeing that particular screen displayed on the app. This phase was performed iteratively such that participants’ survey responses elicited subsequent modifications in the wireframe images, which led to a revised survey design capturing features of the updated wireframe images. A total of 4 consecutive versions of the CPAP tracking module and survey were produced and presented to participants based on the prior participants’ feedback. Thus, during phase 2, participants completed a version of this iterative survey. Design decisions were made based on the participants’ feedback and survey data, and a new CPAP module was developed for Android smartphones. Participants were compensated US $100 for participation.

In phase 3 (mean age 55, SD 8 years), patients who were newly diagnosed with OSA at the University of Chicago Sleep Disorders Clinic, who were CPAP naive, and who owned an Android smartphone were recruited after obtaining informed consent. This phase involved in-field testing of the CPAP tracking module of the app while participants were using a newly prescribed ResMed autoadjusting CPAP machine (AirSense 10 AutoSet), Fitbit Aria scale, and Fitbit. The app combined the new CPAP module with weight loss tracking features to enable participants to experience and evaluate the integrated app. The home screen of the CPAP module graphically depicted CPAP adherence as CPAP use relative to the time spent in bed, expressed as a percentage. The home screen also displayed CPAP use and time spent in bed separately in hours and minutes. An additional page in the app displayed CPAP adherence over a time frame of weeks or months as well as details of daily adherence and mask leak. Each participant was provided with a ResMed AirSense 10 AutoSet CPAP device, Fitbit Aria scale, and Fitbit activity monitor (Fitbit Inspire HR) to use for 3 to 4 weeks. Fitbit data were used to track the time spent in bed, and ResMed data were used to track CPAP wear time to allow calculation of percentage of CPAP adherence relative to the time spent in bed. The participants also received weekly phone calls to troubleshoot any CPAP-, Fitbit-, or app-related issues. In addition, some participants in the later part of the testing received push notifications within an hour of their self-reported bedtime as a reminder to wear CPAP device in the evening. Moreover, a message was sent upon Fitbit sensing waking to remind the participant to wear and synchronize the Fitbit device. Upon completion of phase 3, participants completed a survey that included the System Usability Scale [30] and questions about their positive and negative feedback on the app’s design and burden of use. Participants were compensated US $230 for participation.

### Ethics Approval

This study was approved by the University of Chicago Institutional Review Board (#19-1446). All participants provided informed consent before the study after a member of the research team explained all details of the study and the participants received satisfactory answers to all of their questions.

## Results

### Overview

A total of 37 patients with OSA (27 men and 10 women) participated in this study. Table 1 summarizes the primary questions and main findings of each study phase.

During phase 1, a total of 7 participants (6 men and 1 woman) were enrolled. All participants found the app easy to use, except for some difficulty in searching for specific foods in the database. All participants also endorsed that they felt confident when using the app. Of the 7 participants, 3 (43%) reported that they did not like the overall design of the app, and all 3 of them attributed their dislike to the nutrition tracking. When asked about the feature they liked best, of the 7 participants, 3 (43%) endorsed nutrition tracking, 3 (43%) endorsed weight tracking, and 2 (29%) endorsed physical activity tracking. None of the participants found the app too difficult to use, reported that they would need help, or stated that it was too complex. None of the participants encountered problems with the connected devices, that is, Fitbit activity tracker (Fitbit Inspire HR) or Fitbit weight scale (Fitbit Aria), and the participants commented that both were “easy to use” and that they liked “how the information from it went straight into the app.” Overall, the participants rated the app on an average of 83 on the 100-point System Usability Scale, a score considered to be indicative of a highly usable system compared with other similar systems from a recent review [30,31].

During phase 2, a total of 21 patients were enrolled. The first 6 participants completed the first version of the survey and provided initial responses to graphical representations (an example shown in Figure 2) of the percentage of CPAP use relative to time spent in bed as well as mask leak indicator. In addition to seeing percentage of adherence, participants also wanted to see details of the time spent in bed and CPAP wear time. They also preferred to have mask leak indicators that were always displayed rather than only when a problem arose. The next 3 participants were shown the representations preferred by the initial 6 participants and were asked questions about their understanding of the information displayed. Moreover, they were shown a wireframe that included AHI in addition to mask leaks. Participants accurately interpreted the meaning of the information displayed by the preferred wireframes and the follow-up behavior they should do in response. However, they were confused about how to interpret AHI. Given this feedback, the next 5 participants were shown 3 options for displaying information related to CPAP goal attainment and 2 options for showing mask leak information without the AHI. Participants correctly interpreted the percentage of success and matched their percentage to a goal. However, their opinions about the best way to display the percentage of time adherent to CPAP wear relative to the time spent in bed were mixed. Participants interpreted leak information correctly, but some had concerns about having a green light indicator for the absence of leak, particularly when it was displayed, in addition to poor CPAP adherence (ie, low percentage of success), which they found confusing. The final 7 participants responded to visuals depicting adherence with and without the CPAP percentage goal indicated and displayed leak information with and without “no leak” text to represent the absence of a problem. Most preferred having no indicator displayed when there was not a problem with leak. Participants preferred seeing the CPAP percentage goal for which they were striving. The results of this phase characterized the Android version of the app delivered in phase 3 of the study.

**Figure 3:**
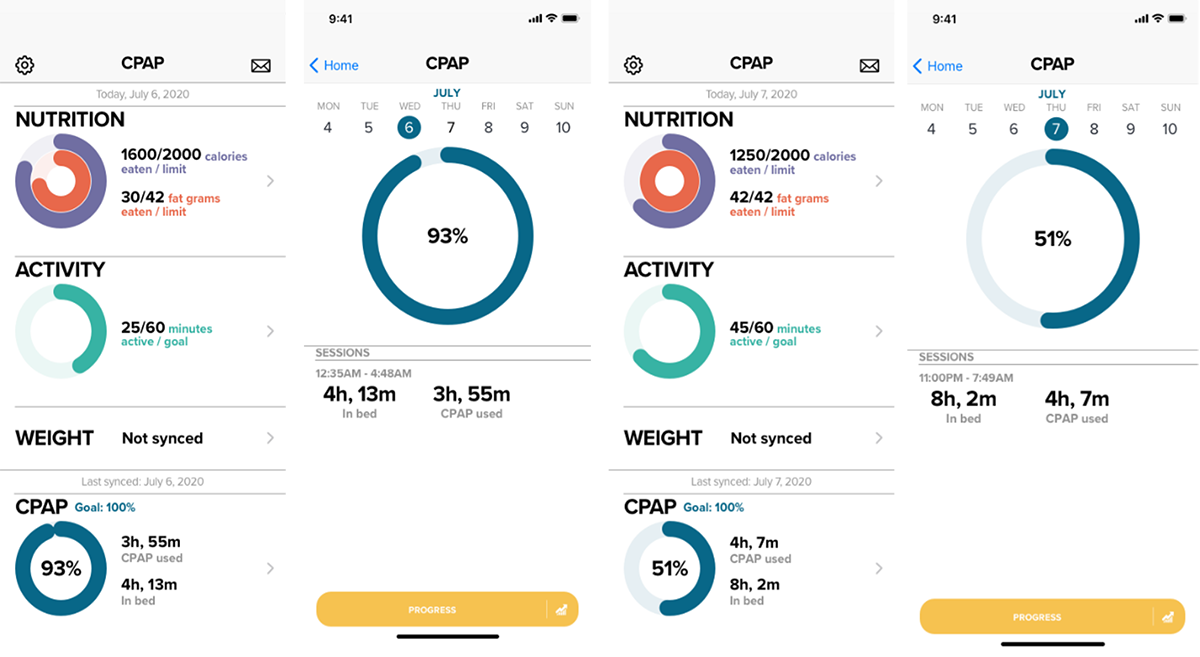
Final app with integrated continuous positive airway pressure, nutrition, activity, and weight tracking features. CPAP: continuous positive airway pressure.

The phase 3 study used app interfaces, as shown in Figure 3. A total of 10 patients with newly diagnosed OSA who were CPAP naive consented to participate. A patient discontinued participation after providing consent, and no data were collected. All 9 participants endorsed that they liked the design and that the app was easy to navigate. Of the 6 users who received push notifications, all found them helpful and well timed. When asked about their favorite features, participants reported a mix of nutrition, physical activity, and CPAP adherence, with half of them endorsing physical activity. When asked about their least favorite features, nutrition, activity, and CPAP adherence were each chosen by 20% (2/10) of the participants. Participants rated the app an average of 80 on the 100-point System Usability Scale, a score considered to be indicative of a highly usable system [31]. Some participants reported during calls that the app interface did not always show 100% CPAP adherence even when they wore their CPAP device for the entire time they spent in bed. After carefully examining our data in response to this feedback, we adjusted the percentage of CPAP adherence calculation logic to allow a 15-minute buffer for the time spent in bed captured by the Fitbit activity tracker. This minor adjustment in our calculation logic protected against a margin of measurement error for time spent in bed as captured by Fitbit [32], while not compromising the accuracy of the percentage of CPAP adherence measure.

**Table 1:** Primary questions and main findings.

Phase	Primary questions	Main findings
1	Are the previously developed weight loss app features and the connected devices usable by the study sample? Are Fitbit activity tracker and Fitbit Aria scale use helpful for tracking their activity and weight, respectively? What design aspects of the weight loss app need improvement?	Rated easy to use by all participants (rated ≥65 on the System Usability Scale); scores ranged 70 to 92.5 out of a possible 100 Helpful and easy to use devices; no issues noted; positive aspect of devices noted by all participants Difficulty searching for specific foods in the database by 4 participants
2	What CPAP^a^ adherence tracking information is helpful to participants? Which graphical displays about CPAP are easy to interpret? Which CPAP tracking features lead to accurate interpretations by participants?	Less information preferable (eg, AHI^b^ not displayed) Color to indicate mask leak only when there is a problem is preferred Clearly displayed 100% success goal is needed Success depicted as percentage of adherence is easily and accurately interpretable
3	Is the new CPAP module integrated with the weight loss app usable and acceptable to participants?	Found to be not burdensome and has an excellent usability rating; all participants rated >65 on the System Usability Scale with a range of 72.5 to 92.5 Request for reminders at appropriate times of day, that is, reminder 1 hour before bedtime to wear a CPAP device and reminder to synchronize Fitbit in the morning upon waking

^a^CPAP: continuous positive airway pressure.

^b^AHI: apnea-hypopnea index.

### Final App Description

The final app design is shown in Figure 3. The app allowed participants to track CPAP adherence, nutrition, physical activity, and weight. The CPAP tracking module showed the percentage of CPAP adherence each night by darkening a portion of a circle’s circumference to represent the percentage of time the CPAP device was used (conveyed by the CPAP device via the AirView API) during the time spent in bed (conveyed by the Fitbit wrist activity monitor). “Goal: 100%” was displayed above the circle to remind participants to wear their CPAP the entire time they spent in bed. The section also displayed, in hours and minutes, the duration of CPAP wear time and time spent in bed separately on the right side of the circle. Thus, CPAP tracking in the final app provided participants with meaningful feedback that aimed to encourage and improve their CPAP adherence. If a participant’s mask had a high leak at any point, the app marked the night with the label “LEAK” and a red dot to alert the participant to troubleshoot mask issues and contact their health care provider, as required. Individualized push notifications approximately 1 hour before the participant’s bedtime reminded participants to use the CPAP device every night and to synchronize their Fitbit every morning upon waking. Notifications were tailored to the participant’s self-reported bedtime for the first week and then adjusted to their average bedtime shown by Fitbit, as data become available.

Nutrition intake was tracked when participants searched for and selected foods in the app or added custom foods or recipes by recording calories and fat gram content. Calories and fat gram intake were calculated every day. Participants were shown daily calorie and fat gram goals within the nutrition section, which were calculated based on their weight. Physical activity was tracked in the app by automatically transferring data from the participants’ wrist-worn Fitbit activity monitor via Bluetooth. Physical activities could also be manually entered by searching the app database, which included a compendium of physical activities rated by their intensity. An activity could be tracked by selecting the specific activity and its duration. Weight was tracked automatically by synchronizing with the Fitbit Aria scale; weight could also be manually entered into the app if needed. For every element tracked by the app, progress over time could be viewed as a weekly or monthly line graph.

Figure 3 illustrates the final app screenshots for a representative participant on 2 consecutive days. As seen, CPAP wear times are quite similar on Wednesday and Thursday nights (3 hours 55 minutes vs 4 hours 7 minutes). On the basis of the current clinical threshold that defines 4 hours of CPAP wear as adherent, this patient would be considered CPAP “nonadherent” on Wednesday night (3 hours 55 minutes of CPAP wear time) and “adherent” on Thursday night (4 hours 7 minutes of CPAP wear time). However, when time spent in bed was considered using our newly developed CPAP tracking module, the patient’s true CPAP adherence relative to the time in spent bed was 93% on Wednesday night and 51% on Thursday night. Thus, our new CPAP adherence metric accounting for time spent in bed provided important and clinically meaningful information about CPAP adherence, which is not captured by current CPAP tracking technologies.

## Discussion

### Principal Findings

We engaged in a 3-phase iterative, user-centered process to develop and test a smartphone app that aimed to support both CPAP adherence and weight loss behaviors in patients with OSA and overweight or obesity. Our user-centered design process identified key information that participants found useful for tracking their adherence to CPAP as well as user interfaces that participants found easy to interpret. We found that participants were not only satisfied with less information about their CPAP use but also that their interpretations were more accurate when less information was provided. In addition, there was a need for explicit descriptions of the information provided, which we accomplished by providing descriptors or comparators such as “Goal: 100%.” The information collected via the surveys supported our design decisions regarding appropriate features, functions, and user interface for the study. Overall, the participants rated the system’s usability as high [31] and reported positive impressions of the app features. In addition, based on participants’ feedback during phase 3, we enhanced the usefulness of the app by adding push notifications to remind users to use their CPAP device (ie, 1 hour before bedtime) and wrist activity monitor (ie, synchronize device every morning) at meaningful times of the day.

### Comparison With Prior Work

A unique feature of our app was the tracking of CPAP use relative to objectively assessed time spent in bed, represented as percentage of CPAP adherence. To harvest the data needed to calculate percentage of CPAP adherence, we leveraged connected technologies, in this case, a ResMed CPAP device (AirSense 10 AutoSet) to track CPAP wear time and a Fitbit wearable activity sensor to track the time spent in bed. This novel CPAP adherence metric provided markedly different information than the available CPAP tracking technologies, which simply report how many hours a CPAP device was used without accounting for the time spent in bed without using a CPAP device. Although OSA can be effectively treated only when CPAP is used during the entire time spent in bed, current clinical guidelines categorize patients as adherent to treatment based on a cutoff point of 4 hours of CPAP use [33]. This adherence definition is widely accepted, but it is primarily based on expert opinion and remains in common use today despite lack of evidence showing that it is sufficient or has any health benefits compared with other more specific measures of use duration [11]. By leveraging wearable sensor technology that can objectively capture time spent in bed, our new app provided a novel, more informative, and clinically meaningful measure of CPAP adherence that can be implemented into clinical guidelines. First, we defined a new CPAP adherence metric that considered both CPAP wear and time spent in bed. Second, our mHealth tool was the first technology to capture this novel percentage of CPAP adherence metric. Thus, the use of the app and the data it provided filled an important gap in the management of OSA for patients and health care providers. Future rigorous research in diverse populations with OSA is warranted using this new CPAP adherence metric and mHealth technology to investigate the role of percentage of CPAP adherence in a variety of patient-centered and clinical outcomes. Future studies can also provide novel insights into the dose-response effect of CPAP adherence on cognitive, cardiovascular, and metabolic outcomes [34].

To date, the effectiveness of eHealth interventions in improving CPAP adherence remains uncertain, highlighting the need for newly designed technology-supported interventions for OSA [19]. As our app fed back information about CPAP use relative to the time spent in bed, it can support patients in reaching their goal of 100% CPAP adherence, that is, wearing their CPAP during the entire time they spent in bed. Real-time app data on percentage of CPAP adherence can be used not only as a self-management tool for patients but also as a monitoring tool for health care providers. Our existing technology platform also allowed for a web-based dashboard to display patient information from the app so that both the patient and provider can see progress toward goals. In addition, the app integrated lifestyle behavior tracking features (diet, physical activity, and body weight) and thus has the potential to enhance self-management and positive behavior change toward weight loss goals in patients with OSA [19]. Similar to our integrated app design, other emerging mHealth technologies target multiple behavior change interventions in the population with OSA [35,36].

The iterative user-centered design process used to develop our final app integrating CPAP tracking has notable strengths [37,38]. A few prior apps designed for populations with OSA relied primarily on the views expressed by clinical experts in focus groups with minimal feedback from patients [35]. By engaging patients in the design process from the outset and throughout, we increased the likelihood that the final app would be easy to use, helpful, and engaging to the targeted end users [37,39,40]. We used low-fidelity wireframes to represent user interfaces and embedded them in surveys to gather feedback quickly, without requiring time-consuming programming. The iterative nature of the surveys allowed us to respond promptly to end-user feedback about features or graphical displays that were not functional, not interpreted correctly, or not liked, bringing us closer to a feasible and acceptable interface. The process allowed our research team to progress by finding features, functions, and interfaces that accurately represented the required information and that satisfied the intended users, before engaging in extensive and costly programming efforts involved in app design.

### Limitations

Our study has several limitations. Although new participants were enrolled in each study phase, resulting in a total sample of 37 that included patients with OSA who were experienced with CPAP use and those who were CPAP naive, the sample was collected from a single center, and a larger, more diverse sample size may provide additional insights and feedback for further improvements and refinements to the app. In this formative work to cocreate an app to support end users, we did not collect demographic characteristics, except for age and gender. Indeed, additional patient characteristics (eg, race or ethnicity, socioeconomic status, and prior CPAP adherence) may affect the usability outcomes of our tool. Thus, as a next step, large studies in diverse patient populations are needed to derive more generalizable assessments of the usability and efficacy of this mHealth tool. In the future, an iOS version of the app will also be needed to increase generalizability.

Our mHealth tool relied on specific manufacturers and used “consumer grade” sleep trackers, which was a pragmatic choice. Although each sleep-tracking device has its own margin of measurement error, the devices and apps are meant to support behavior as part of an intervention rather than for diagnostic validity. Notably, the research-grade devices (eg, Actigraphy) do not allow people to receive real-time feedback on their behavior from the device, which is critical for self-monitoring and intervention success. Although absolute values may have some margin of error depending on the tracking device, they can still reliably assess trends over time for a given individual. Moreover, in our study, a recent generation Fitbit model (ie, Fitbit Inspire HR) was used, which performs better than the early generation models (owing to the addition of heart rate into the algorithm), especially in differentiating wake from sleep [24]. Nevertheless, over time, improvements and updates will need to be made to our algorithm to keep pace with the rapidly changing technology. Our final CPAP module does not display “AHI” based on feedback from participants who were confused about how to interpret it and preferred less information to be displayed. However, misinterpretation of AHI is possible. In future versions of the app, an AHI metric, that is, apnea burden during the “off-CPAP time,” could be displayed to track treatment effectiveness [41]. Such an additional feature could potentially foster patient adherence, which warrants further rigorous testing in larger samples.

In this study, we opted to measure the percentage of CPAP adherence based on the time spent in bed captured by Fitbit and not the actual time spent asleep, which may appear as a potential limitation. CPAP use would ideally be required during the entire sleep period. However, CPAP adherence displayed as a percentage of time spent in bed is more meaningful for patients (ie, end users) in meeting the goal of CPAP use during all sleep periods occurring over time spent in bed. In addition, there is evidence to suggest that Fitbit activity trackers have acceptable levels of measurement accuracy for the time spent in bed compared with research-grade accelerosensors but may either overestimate or underestimate the actual sleep duration depending on the selected sleep-mode setting [24,32]. Finally, it is noteworthy that testing the efficacy of the app was beyond the scope of this study; thus, future trials are necessary.

### Conclusions

We developed a new mHealth tool that filled a significant gap in the clinical management of patients with OSA. Our app used a novel CPAP adherence metric, that is, percentage of CPAP adherence that measures CPAP use relative to the time spent in bed and allows tracking of lifestyle behaviors targeting weight loss, such as diet, physical activity, and weight, for use in the population with OSA and comorbid overweight or obesity. The newly developed mHealth technology allowed tracking of both CPAP adherence and lifestyle behaviors, giving it the potential to support multiple behavior changes that optimize care for patients with OSA.

## Abbreviations

AHI: apnea-hypopnea index
API: application programming interface
CPAP: continuous positive airway pressure
mHealth: mobile health
OSA: obstructive sleep apnea
